# Non-employment and low educational level as risk factors for inequitable treatment and mortality in heart failure: a population-based cohort study of register data

**DOI:** 10.1186/s12889-021-10919-1

**Published:** 2021-06-02

**Authors:** Anna Ohlsson, Nils Eckerdal, Bertil Lindahl, Marianne Hanning, Ragnar Westerling

**Affiliations:** 1 Department of Public Health and Caring Sciences, Uppsala University, Box 564, 751 22 Uppsala, Sweden; 2 Department of Statistics, Uppsala University, Box 513, 751 20 Uppsala, Sweden; 3 Department of Medical Sciences, Uppsala University, 751 85 Uppsala, Sweden

**Keywords:** Heart failure, Renin-angiotensin system blockers, Equity in health care, Employment status, Educational level

## Abstract

**Background:**

The risk of heart failure is disproportionately high among the socioeconomically disadvantaged. Furthermore, socioeconomically deprived patients are at risk of inequitable access to heart failure treatment and poor outcomes. Non-employment as a risk factor in this respect has not previously been studied at the level of the individual. The aim of this register-based cohort study was to analyse equity in access to renin-angiotensin system blockers and mortality, by employment status and educational level.

**Methods:**

The study population consisted of Swedish patients aged 20–64 years hospitalised for heart failure in July 2006–December 2010, without a heart failure hospitalisation within one year or more before index hospitalisation and without renin-angiotensin system blocker dispensation in the 6 months preceding index hospitalisation. Non-access to renin-angiotensin system blockers, measured as drug dispensations, was investigated by employment status and educational level through logistic regression. Cox regression models were used to obtain hazard ratios for all-cause death by educational level and employment status. Interaction analysis was used to test whether associations between access to treatment and mortality differed by employment status.

**Results:**

Among the 3874 patients, 1239 (32%) were women. The median age was 57 years. Fifty-three percent were employed. The non-employed patients had more comorbidity and lower access (68%) to renin-angiotensin system blockers compared with the employed (82%). The adjusted odds ratio for non-access to renin-angiotensin system blockers among the non-employed was 1.76. Non-employment was associated with an adjusted hazard ratio of 1.76 for death. Low educational level was associated with a higher death risk. Mortality was highest among the non-employed without access to renin-angiotensin system blockers and the association between access to renin-angiotensin system blockers and survival was slightly weaker in this group.

**Conclusions:**

Non-employment and low educational level were associated with elevated mortality in heart failure. Non-employment was a risk factor for lower access to evidence-based treatment, and among the non-employed access to treatment was associated with a slightly smaller risk reduction than among the employed. The results underscore that clinicians need to be aware of the importance of socioeconomic factors in heart failure care.

**Supplementary Information:**

The online version contains supplementary material available at 10.1186/s12889-021-10919-1.

## Background

Heart failure (HF) is an increasing health problem globally [1]. HF incidence is disproportionately high among the socioeconomically disadvantaged and outcomes are poorer in this group [2–6]. Furthermore, there are indications of socioeconomic inequity in access to HF care, including evidence-based pharmacological HF treatment [7, 8].

One of the mortality-reducing evidence-based treatments in HF with reduced ejection fraction (HFrEF) is renin-angiotensin system blockers (RASb). Along with beta-blocker therapy, RASb have long been recommended in clinical guidelines as a first-line treatment for HFrEF. Thus, most HFrEF patients should receive such treatment. For HF with preserved ejection fraction (HFpEF), there is no evidence-based mortality-reducing medication.

Socioeconomic position has often been defined using occupation, and classified based on e.g., the degree of manual work and/or the amount of job control [9, 10]. Occupational class is associated with health and mortality outcomes in a range of health problems [11], including HF [4]. Several other socioeconomic indicators, such as educational level and income, are also strongly associated with health outcomes [11]. However, according to Swedish data, avoidable mortality (preventable by health policy or health care measures) may differ more between those working and those not working than between different occupational classes [12]. Non-employment has been linked to low health care utilisation relative to perceived need [13]. In a previous study, we found that non-employed patients hospitalised for HF were at higher risk than those employed of not getting access to treatment with RASb [7].

Socioeconomic deprivation is associated with higher HF mortality [2, 3, 6]. Previous investigators have mainly studied education and income, or aggregated measures, and socioeconomic indicators have often been studied by area, rather than by individual [2, 3, 6, 14]. Studies investigating individual employment status in relation to HF mortality are lacking. Furthermore, equity in access to RASb treatment by socioeconomic factors is incompletely studied, and results are conflicting [5–7, 14, 15].

We aimed to study RASb access and mortality by educational level and employment status, in a population register cohort of hospitalised HF patients of working age, and to analyse possible excess mortality among non-employed patients without access to RASb. We tested 1) whether a low educational level or non-employment was associated with non-exposure to RASb; 2) whether a low educational level or non-employment was associated with a higher risk of all-cause death; and 3) whether non-employment was associated with additional mortality risk due to interaction with non-exposure to RASb.

## Methods

### Data

We used individual-level register data linked by unique personal identity numbers, from several Swedish total population registers: hospitalisation data from the National Patient Register [16]; drug dispensations from the Prescribed Drug Register [17], cause of death from the Cause of Death Register [18]; and sociodemographic data from the Longitudinal Integration Database for Health Insurance and Labour Market Studies (“LISA” by its Swedish acronym) [19]. Coverage is > 99% for both inpatient care in the National Patient Register and drug dispensations in the Prescribed Drug Register. The Cause of Death Register contains all deaths among persons registered in the Swedish national registration at the time of their death. In LISA, coverage differs between variables. In this study, there were 1% missing data for educational level and 2.8% missing data for employment status. Missingness is reported by presenting the number actually analysed for each variable in the results section.

### Study population

**Fig. 1:**
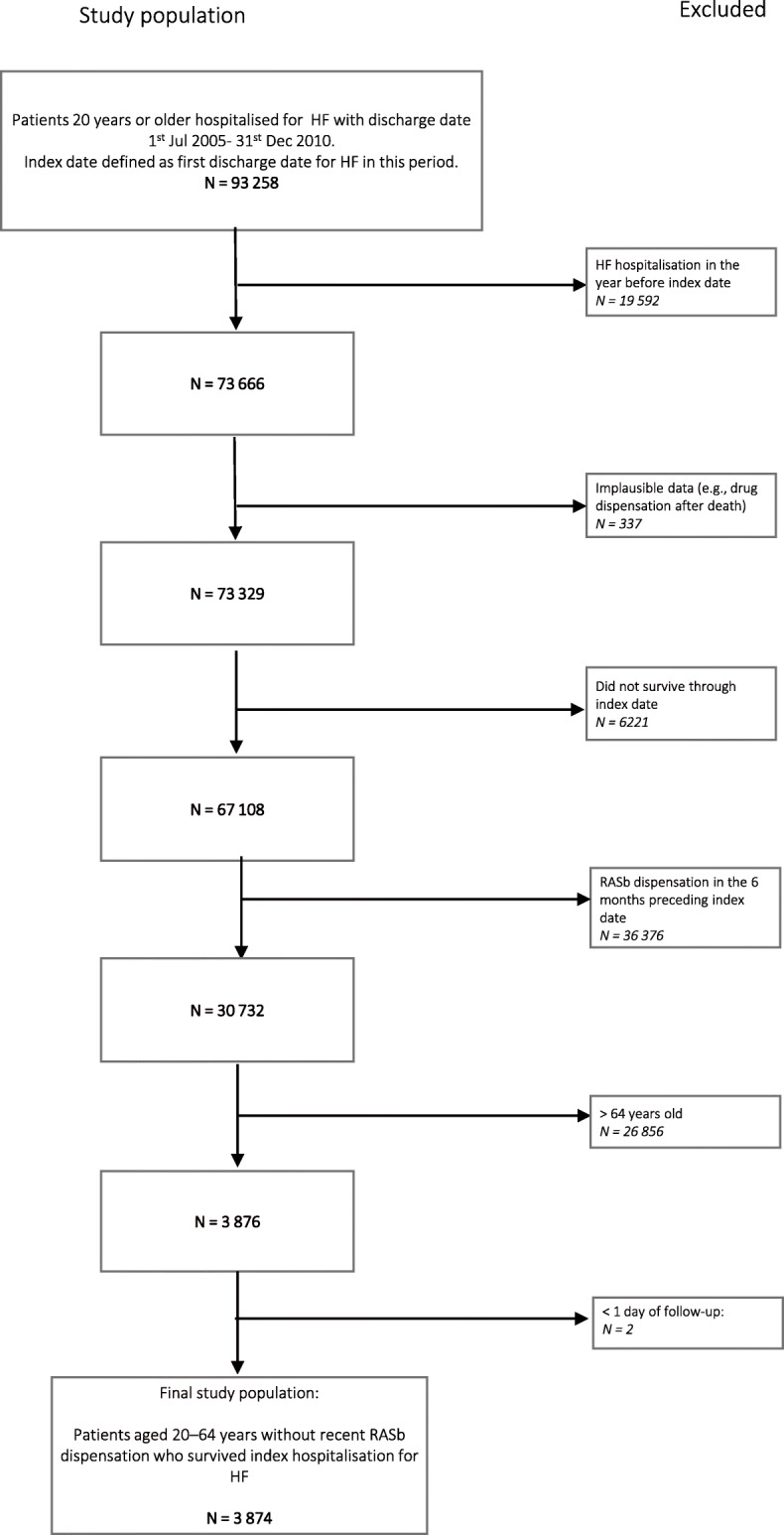
Selection of study population

The study population comprised all patients aged 20–64 years who survived an index HF^1^ hospitalisation in the period 1st July 2006–31st December 2010, without a previous HF hospitalisation within one year or more before index hospitalisation and without RASb dispensation in the 6 months preceding index hospitalisation. The index hospitalisation was the first hospitalisation in the study period for each individual, and the index date was the discharge date for that hospitalisation. Figure 1 depicts the selection of the study population, which consisted of 3874 individuals.

### Outcomes

For analyses of the associations between low educational level or non-employment and non-exposure to RASb, the outcome was defined as at least one dispensation of any dose of either an angiotensin-converting enzyme inhibitor (ACEI) or an angiotensin receptor blocker (ARB)^2^ during follow-up, for those surviving 30 days or more. For analyses of the associations of low educational level or non-employment with all-cause death, and the association of non-employment with additional mortality risk due to interaction with non-exposure to RASb, the outcome was death from any cause during follow-up.

### Exposures

#### Renin-angiotensin system blockers

For survival analysis, exposure to RASb was defined as at least one dispensation of either an ACEI or an ARB at any time during follow-up. Using the date for the first dispensation after index hospitalisation, we calculated a time-dependent exposure variable for RASb, with time between index date and dispensation designated as unexposed, and time from dispensation until end of follow-up designated as exposed.

#### Employment status

Employment status was extracted from a LISA variable, gainful employment status, based on a) the presence of an income statement in November of the year in question, and b) a minimum yearly income (different levels by year, age and sex). We used the value for employment in the year before index hospitalisation. The original categorisation in LISA was:

- Gainfully employed: income statement from gainful employment in November the year in question & income above the limit to be classified as continuously employed.
- Not continuously employed: income statement from gainful employment in November the year in question & income below the limit to be classified as continuously employed.
- Not employed: no income statement from gainful employment in November the year in question.

We collapsed the two latter categories and dichotomised the variable into either employed or non-employed.

#### Education

The highest attained educational level in the year before index for each individual was retrieved from LISA, where it was divided into 7 categories. We re-categorised education as compulsory school (≤ 9 years, upper secondary school (approximately 11–12 years), or post-secondary school (approximately ≥12 years).

### Other covariates

#### Comorbidity

Comorbidity was defined based on ICD-10 diagnoses (see Additional file 1, S Table 1) at any inpatient hospitalisation within 1.5 years before and including the index hospitalisation.

For the analysis of non-exposure to RASb, we adjusted for hypertension, angina pectoris, myocardial infarction, coronary artery bypass grafting (CABG), renal dysfunction, diabetes mellitus, psychiatric disease, and dementia.

For survival analyses, we adjusted for hypertension, angina pectoris, myocardial infarction, atrial fibrillation/flutter, pacemaker, CABG, stroke, peripheral vascular disease, lung disease, renal dysfunction, diabetes mellitus, anaemia, dementia, cancer, liver disease, rheumatic disease, and psychiatric disease.

#### Hospitalisation-free time

The study entry time varied. The time free from hospitalisation before inclusion, i.e., between 1st July 2006 and index hospitalisation, was calculated for each individual and adjusted for.

#### Other medication

Beta-blockers or aldosterone antagonists, also guideline-recommended treatments for HF, were taken into account as separate covariates. Individuals were defined as getting these therapies if they had at least one dispensation of either drug within one year after index hospitalisation.

### Statistical analysis

Logistic regression was used to model the associations between low educational level and non-employment respectively, with non-exposure to RASb within 30 days, among those surviving at least 30 days. A multivariable model was furthermore adjusted for age, gender, comorbidity, other medication, and index year in addition to the covariates educational level and employment status. We also performed this analysis including all patients and defining RASb exposure as dispensation at any time during follow-up, as a sensitivity analysis.

Cox regression was used to model the associations between RASb exposure, low educational level, non-employment, and all-cause death. The multivariable model was adjusted for age, gender, hospitalisation-free time, comorbidity, and other medication.

The assumption of proportional hazards was examined using univariate score tests and found to be satisfied in most cases, but not for the covariates RASb, beta-blocker or aldosterone antagonist in the fully adjusted model (model 5). Consequently, the Cox model would estimate an average hazard ratio (HR) across time from study inclusion for these variables. However, since the efficiency of RASb is well-studied, the time variations of the HR for RASb observed here were not investigated further.

We assessed multiplicative interaction between employment and RASb exposure by adding an interaction term to the Cox regression model, and by calculating the HR for each exposure category. Interaction was also assessed on the additive scale by calculating the relative excess risk due to interaction (RERI) [20].

#### Additional analyses

To examine the proportion of external causes among deaths, we tabulated external causes of death against employment status.

We examined the sensitivity of our results to underreporting of renal dysfunction by recoding a series of percentages of renal dysfunction non-cases into cases and running the Cox regression models under these conditions. This was repeated 10,000 times for each percentage. We also compared the hospitalisation-free time before study entry between the employed and non-employed, using the chi-square test.

## Results

### Characteristics

As presented in Table 1, the overall median age was 57 years. The study cohort included 1239 (32%) women. About half of the patients had upper secondary school as their highest educational level, and around 20% had finished post-secondary school. Fifty-three percent were employed. Women were overrepresented among those with higher education and underrepresented among those with employment. The overall median yearly income was €13,673.^3^ The median follow-up time for the entire cohort was 737 days. Hypertension (28%) and atrial fibrillation/flutter (25%) were the two most common comorbidities, followed by diabetes mellitus, lung disease, and myocardial infarction. Psychiatric disease was found in 9% of patients. Non-employed patients had higher rates of all comorbidities except cancer. Those with the lowest educational level had somewhat more comorbidities than those with the highest educational level. Among the 3731 patients surviving at least 30 days, RASb were dispensed to 2802 patients (75%) during follow-up. Among the employed, 82% were dispensed RASb, compared with 68% among the non-employed (Table 1). Patient characteristics by RASb dispensation within 30 days, among 30-day survivors, are provided in S Table 2, in additional file 2.

**Table 1:** Patient characteristics by employment status and educational level

Total (%)		Distribution (%)				
	Total cohort	Employed	Non-employed	Post-secondary school	Upper secondary school	Compulsory school
	*N* = 3874	*N* = 3836 (with data on employment)		*N* = 3767 (with data on education)		
		2046 (53.3)	1790 (46.7)	719 (19.1)	1818 (48.3)	1230 (32.7)
Age (years)						
Median	57	56	58	56	57	58
25th percentile	50	48	52	47	48	52
75th percentile	61	60	62	61	61	62
Gender						
Women	1239 (32.0)	585 (28.6)	648 (36.2)	263 (36.6)	583 (32.1)	366 (29.8)
Men	2635 (68.0)	1461 (71.4)	1142 (63.8)	456 (63.4)	1235 (67.9)	864 (70.2)
Income (€)^a^						
Median	13,673	17,490	11,240	17,279	13,808	12,356
25th percentile	10,308	12,769	8817	11,548	10,587	9760
75th percentile	19,962	23,466	14,000	24,365	19,548	18,346
Follow -up time (days)						
Range	1–1643	1–1641	1–1643	1–1635	1–1639	1–1643
Median	737	777	688	738	726	746
25th percentile	322	372	281	307	333	312
75th percentile	1179	1207	1151	1135	1178	1192
Comorbidity						
Hypertension	1088 (28.4)	576 (28.2)	512 (28.6)	200 (27.8)	521 (28.7)	339 (27.6)
Diabetes mellitus	585 (15.1)	198 (9.7)	377 (21.1)	97 (13.5)	267 (14.7)	196 (15.9)
Angina pectoris	213 (5.5)	93 (4.5)	117 (6.5)	34 (4.7)	103 (5.7)	68 (5.5)
Myocardial infarction	424 (10.9)	193 (9.4)	225 (12.6)	62 (8.6)	206 (11.3)	143 (11.6)
Atrial fibrillation/flutter	958 (24.7)	517 (25.3)	429 (24.0)	176 (24.5)	434 (23.9)	323 (26.3)
Pacemaker	115 (3.0)	56 (2.7)	58 (3.2)	28 (3.9)	49 (2.7)	34 (2.8)
Stroke	120 (3.1)	36 (1.8)	84 (4.7)	17 (2.4)	59 (3.2)	42 (3.4)
Renal dysfunction	208 (5.4)	69 (3.4)	137 (7.7)	29 (4.0)	107 (5.9)	67 (5.4)
Vascular disease	65 (1.7)	25 (1.2)	40 (2.2)	8 (1.1)	31 (1.7)	25 (2.0)
Rheumatic disease	65 (1.7)	20 (1.0)	45 (2.5)	10 (1.4)	28 (1.5)	27 (2.2)
Lung disease	581 (15.0)	198 (9.7)	378 (21.1)	71 (9.9)	283 (15.6)	207 (16.8)
Liver disease	92 (2.4)	33 (1.6)	59 (3.3)	13 (1.8)	45 (2.5)	32 (2.6)
CABG	193 (5.0)	80 (3.9)	111 (6.2)	31 (4.3)	94 (5.2)	62 (5.0)
Anaemia	176 (4.5)	44 (2.2)	131 (7.3)	23 (3.2)	91 (5.0)	58 (4.7)
Cancer	118 (3.0)	64 (3.1)	53 (3.0)	22 (3.1)	50 (2.8)	44 (3.6)
Dementia	8 (0.2)	3 (0.1)	5 (0.3)	1 (0.1)	5 (0.3)	2 (0.2)
Psychiatric disease	359 (9.3)	97 (4.7)	257 (14.4)	43 (6.0)	171 (9.4)	137 (11.1)
RASb < 30 days (among 30-day survivors, *N* = 3731)	2802 (75.1)	1627 (82.2)	1157 (67.5)	524 (75.0)	1327 (76.0)	886 (75.0)

RASb, Renin-angiotensin system blockers

CABG, coronary artery bypass grafting

a) Converted from Swedish krona; rate from the Swedish central bank (Riksbanken); https://www.riksbank.se/sv/statistik/ accessed 14 September 2020

### Treatment

Among those surviving 30 days or more, non-employment was associated with a crude odds ratio (OR) of 2.22 (95% confidence interval 1.90, 2.59) for not being dispensed RASb within 30 days of index date (Table 2). The adjusted OR was 1.76 (1.47, 2.11). Other covariates associated with non-exposure to RASb were female gender and lower age. Educational level was not associated with exposure to RASb. Estimates were very similar for the total cohort and when defining RASb exposure as dispensation at any time during follow-up.

**Table 2:** Odds ratios (ORs) for non-dispensation of renin-angiotensin system blockers (RASb) within 30 days, among 30-day survivors.

*N = 3731*	Crude OR	OR adjusted for age	OR adjusted for age and gender	Multivariable model including: employment, education, age^a^, gender, comorbidity^b^, other medication^c^, and index year.
Employment *(N = 3694)*				
Employed	Ref	Ref	Ref	Ref
Non-employed	2.22 (1.90, 2.59)	2.28 (1.95, 2.66)	2.20 (1.88, 2.57)	1.76 (1.47, 2.11)
Education *(N = 3628)*				
Compulsory school	1.00 (0.81, 1.24)	1.02 (0.82, 1.27)	1.07 (0.86, 1.34)	0.86 (0.67, 1.10)
Upper secondary school	0.95 (0.77, 1.16)	0.95 (0.78, 1.17)	0.99 (0.80, 1.21)	0.84 (0.67, 1.06)
Post-secondary school	Ref	Ref	Ref	Ref
Age (years)	0.99 (0.98, 1.00)			0.99 (0.98, 1.00)
Female gender	1.99 (1.70, 2.32)	1.98 (1.69, 2.30)		1.76 (1.47, 2.11)

^a^Age as continuous variable

^b^Comorbidity with: hypertension, angina pectoris, myocardial infarction, coronary artery bypass grafting, renal dysfunction, diabetes mellitus, dementia, psychiatric disease.

^c^Beta-blocker or aldosterone antagonist.

### Mortality

**Fig. 2:**
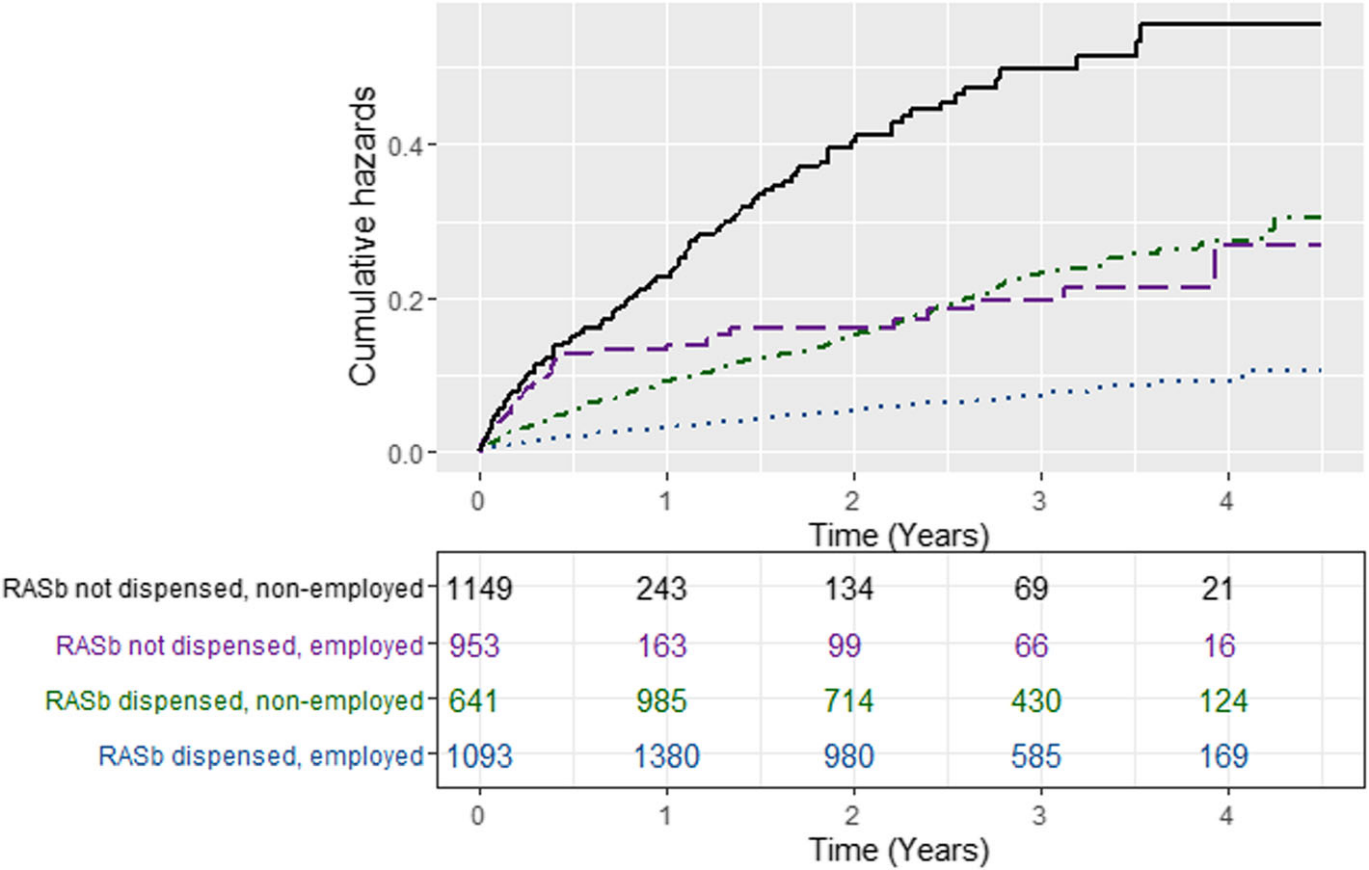
Unadjusted cumulative hazard curves for death from all causes, by exposure group. Legend: Cumulative hazard curves for the four groups included in the accompanying table. The table shows group sizes over time. A steep slope indicates a period of high mortality rate

There were 501 (13%) deaths from any cause among patients included in the multivariable Cox regression analyses. The one-year mortality was 7.6% overall and 11.2% among the non-employed (data not shown). Figure 2 depicts unadjusted cumulative hazards in the different patient groups, showing an excess mortality among the non-employed patients not dispensed RASb. As presented in Table 3, non-exposure to RASb was associated with a crude HR of 3.06 (2.55, 3.68) for death and non-employment was associated with a crude HR of 2.86 (2.36, 3.45) for death. Lower vs higher educational level and higher vs lower age were also associated with higher crude HRs for death. Adjustments for age (model 2) and gender (model 3) scarcely changed the estimates. The estimates were attenuated in model 4 (including educational level, non-employment, hospitalisation-free time, comorbidity, and other medication), so that the overall HR for death associated with non-exposure to RASb was 1.66 (1.33, 2.06), and that associated with non-employment was 1.76 (1.43, 2.17). Lower education remained significantly associated with a higher HR for death, and male gender became significantly associated with a higher HR for death in model 4.

**Table 3:** Hazard ratios (HRs) for all-cause death

*N = 3874*	Crude HR	Model 2 HR adjusted for age	Model 3 HR adjusted for age and gender	Model 4 Multivariable model including: employment, education, hospitalisation-free time, comorbidity^a^, other medication^b^.	Model 5 Multivariate model including: employment, education, hospitalisation-free time, comorbidity^a^, other medication^b^, and interaction: no RASb*non-employment
No RASb dispensed	3.06 (2.55, 3.68)	3.23 (2.68, 3.89)	3.25 (2.69, 3.92)	1.66 (1.33, 2.06)	2.86 (2.00, 4.09)
Employment (*N = 3836*)					
Employed	Ref	Ref	Ref	Ref	Ref
Non-employed	2.86 (2.36, 3.45)	2.70 (2.23, 3.27)	2.69 (2.22, 3.26)	1.76 (1.43, 2.17)	2.21 (1.73, 2.84)
Education (*N = 3767*)					
Compulsory school	1.81 (1.36, 2.42)	1.67 (1.25, 2.23)	1.67 (1.25, 2.23)	1.37 (1.02, 1.84)	1.39 (1.03, 1.86)
Upper secondary school	1.61 (1.21, 2.13)	1.61 (1.22, 2.13)	1.61 (1.22, 2.13)	1.38 (1.04, 1.84)	1.40 (1.06, 1.86)
Post-secondary school	Ref	Ref	Ref	Ref	Ref
Age	1.05 (1.04, 1.06)			1.03 (1.02, 1.05)	1.03 (1.02, 1.05)
Male gender	0.86 (0.72, 1.03)	0.87 (0.72, 1.04)		1.24 (1.02, 1.51)	1.23 (1.01, 1.50)
Interaction: No RASb*non-employment *(N = 3836)*					0.47 (0.31, 0.70)

RASb, Renin-angiotensin system blockers

a) Comorbidity with: hypertension, angina pectoris, myocardial infarction, atrial fibrillation/flutter, pacemaker, coronary artery bypass grafting (CABG), stroke, peripheral vascular disease, lung disease, renal dysfunction, diabetes mellitus, anaemia, dementia, cancer, liver disease, rheumatic disease, psychiatric disease

b) Beta-blocker or aldosterone antagonist

In model 5, we added interaction terms to assess multiplicative interaction between non-exposure to RASb and non-employment. There was significant negative interaction between RASb exposure and employment status, reflecting a weaker association between RASb exposure and death in the non-employed. In model 5, the HR for death for those non-employed with RASb exposure was 2.21 (1.73, 2.84). For the employed without RASb exposure, the HR for death was 2.86 (2.00, 4.09) (Table 3). We also calculated the HR for death among those non-employed and without access to RASb (compared with those employed with access to RASb) and it was 2.96 (2.19, 4.00) (Table 4). The RERI for RASb and employment was calculated, to assess interaction on the additive scale, and found to be − 1.16 (− 2.48, 0.25), i.e., not significant.

**Table 4:** Adjusted hazard ratios for categories of RASb exposure and employment, with the reference employment & RASb (confidence intervals in parentheses).

		RASb	
		No	Yes
Employment	No	2.96	2.21
		(2.19, 4.00)	(1.73, 2.84)
	Yes	2.86	1
		(2.00, 4.09)	(Ref)

RASb, Renin-angiotensin system blockers

Adjustments for: age; gender; hospitalisation-free time; comorbidity with: hypertension, angina pectoris, myocardial infarction, atrial fibrillation/flutter, pacemaker, coronary artery bypass grafting (CABG), stroke, peripheral vascular disease, lung disease, renal dysfunction, diabetes mellitus, anaemia, dementia, cancer, liver disease, rheumatic disease, psychiatric disease; beta-blocker or aldosterone antagonist therapy

The sensitivity analysis for renal dysfunction showed that underreporting would not substantially affect the HRs for RASb non-exposure, employment, or their interaction. We did not find a higher proportion of external causes among the non-employed.

Hospitalisation-free time did not differ significantly between the employed and non-employed.

## Discussion

The main findings of this study were lower access to RASb for the non-employed HF patients, higher mortality for the non-employed and those with low educational level, and a somewhat weaker association between RASb exposure and survival for the non-employed compared with the employed.

### Access to RASb

The adjusted OR of 1.76 for the association between non-exposure to RASb and non-employment is noteworthy. To our knowledge, no other investigators have analysed access to RASb by employment status. In a Dutch primary care population where individual-level socioeconomic status was self-reported and defined mainly by occupation, triple treatment (i.e., diuretics, RASb, and beta-blocker) and beta-blocker treatment were to a greater extent prescribed to those with higher socioeconomic status. Several other studies found no differences in RASb access by socioeconomic factors [6, 14, 15].

We defined access to treatment as being dispensed RASb at a pharmacy. Thus, lower access for the non-employed could have two main causes; 1) less treatment was prescribed to this group, or 2) the non-employed were less likely to collect treatments prescribed.

Non-prescription could be medically motivated in case of medication intolerance or contraindications for RASb (e.g., worsening renal dysfunction during RASb treatment); due to HFpEF, where RASb are neither effective nor recommended; or due to prescribers’ bias against non-employed patients, leading to substandard treatment.

In the present study, we adjusted for comorbidity that affected the chance of receiving a RASb prescription (e.g., renal dysfunction and hypertension). Although data on type of HF were lacking, there is no plausible reason that HFpEF would be more common in the non-employed and justify less RASb prescription. Previous studies have shown that care providers’ bias against disadvantaged patients may contribute to health disparities [21]. Such bias could explain some of our findings.

Possible reasons for non-employed patients to refrain from collecting prescribed drugs include financial constraints, psychiatric morbidity, and low health literacy. A low health literacy, i.e., limited “knowledge, motivation and competencies of accessing, understanding, appraising and applying information to form judgment and make decisions concerning healthcare, disease prevention and health promotion” [22], has been shown to influence medication adherence in HF [23, 24]. Health literacy is associated with socioeconomic factors, mainly educational attainment, and with worse health, such as poorer blood pressure control [25].

Furthermore, depression or other psychiatric morbidity, including substance abuse, might coexist with non-employment, and contribute to lower motivation and less resources to maintain health. We attempted to account for this by adjusting for psychiatric diagnoses.

Although we do not know the exact reasons, the non-employed hadlower access to RASb, which possibly reflects inequitable treatment of this patient group.

### Mortality

The overall risk for all-cause death was considerably higher (HR 1.76) for the non-employed HF patients than for those employed, and higher for those with up to secondary vs post-secondary education, even after adjustments.

In the general population, unemployment is associated with higher all-cause and cause-specific mortality [26–29]. Although such associations may be the result of so-called health selection into employment [30], there is support for at least a partial causal effect of unemployment on mortality [27, 31, 32]. Possible mechanisms linking unemployment to health outcomes include financial strain, psychological health effects, social norms and stigmatisation, and unhealthy behaviours [33–35]. The relationships between mortality and other socioeconomic indicators, e.g., education and income, are also well-established [11, 36]. In our sample, the employment rate (53%) was relatively low, compared with the general Swedish population (age 15–74 years) where employment in 2005–2010 was around 65% overall: 63% among women and 67% among men [37]. In our data, 33% had only compulsory education, compared with 20% in the general population, and 20% had post-secondary education, compared with around 35% in the general population. The median income in our data (over the entire study period) was only around 63% of the Swedish median for 2005. Interestingly, in our cohort, women were under-represented among the employed, but over-represented among those with post-secondary education. These relationships mirror those in the general population [37], and are noteworthy considering that the median income for those employed in our sample was slightly higher than that for those with post-secondary education.

In HF, other investigators have also found higher mortality for some socioeconomic measures, such as income [38, 39], or composite measures [2, 3, 40]. Such increases in mortality have been associated with comorbid conditions or unhealthy behaviours. For example, Witte et al. found that non-cardiovascular hospitalisation, not HF symptoms or access to therapy, explained the higher mortality associated with socioeconomic deprivation in a UK cohort of outpatients treated in cardiology clinics [2]. However, another UK study by Lawson and colleagues concluded that comorbidities and lifestyle factors did not fully explain the higher mortality in the socioeconomically deprived group, and that the focus should be on health care and social interventions to improve equity [3]. In a Catalonian HF population, lower income was independently associated with higher mortality and lower access to specialised care, and the researchers highlighted the need for tailored health care management for patients with low socioeconomic status [38].

Cardiovascular morbidity and mortality are associated with a number of lifestyle factors, such as smoking, alcohol consumption, and diet, and with obesity. Data on these factors were not available in this study, but it is possible that they contributed to higher mortality among the non-employed. Nearly all of the measured comorbidities in this study were more common among the non-employed, particularly diabetes mellitus, lung disease, and psychiatric disease. Notably, diabetes mellitus type 2 is closely associated with obesity, and lung disease is more prevalent in smokers. Although the differences in comorbidity are relevant and consistent with previous research on other socioeconomic factors, they did not account for all of the differences in HRs for death in our data.

Thus, in contrast to the conclusions by Witte et al. [2], our results prompt the question of whether lower access to treatment is in fact part of the reason for the higher mortality among the non-employed in this hospitalised Swedish cohort, and whether the Swedish health care system is delivering equitable HF care.

### Interaction analysis

The non-employed patients in our cohort were dispensed less RASb and had the highest mortality of the studied groups.

The unadjusted cumulative hazard was highest in the non-exposed and non-employed group and lowest in the exposed and employed (Fig. 1).

The weaker association between RASb access and survival among the non-employed in our study, although small in magnitude, was statistically significant and possibly clinically relevant. There is no expected biological difference due to non-employment per se, that would explain a lower effectiveness. Thus, such a difference in the association of RASb with survival would more likely be related to lifestyle factors or comorbidity associated with non-employment.

The likelihood of actual intake of dispensed drugs may differ between groups, as may the propensity to repeatedly collect drugs following a first dispensation. Again, health literacy or psychiatric morbidity may affect both these aspects of drug adherence. Health literacy has been found to mediate the relationship between subjective social status and depressive symptoms among HF patients [41]. According to an overview of systematic reviews, medication adherence in chronic diseases was negatively impacted by depression, and might be greater in those with higher socioeconomic status and employment [42].

Alternatively, the weaker association between RASb and survival could be due to causes of death neither affected by RASb nor related to measured comorbidity. Non-cardiovascular hospitalisation and mortality explained the higher mortality associated with low socioeconomic status in a HF cohort in UK [2]. Furthermore, in a study by our research group, unemployment was associated with higher mortality from external causes, particularly suicides [43]. We did not find any such association between employment and external causes of death in the present data, although this could be due to a very small number of external deaths: only 2.5% of all deaths.

### Strengths and limitations

To our knowledge, this study is the first in which total population individual-level data on employment status and educational level were used to analyse these socioeconomic factors in relation to access to treatment and mortality. The Swedish health care and demographic population registers used are of high quality.

However, because of the observational study design, we could not confirm causality or refute residual confounding.

A specific limitation of this study, due to the nature of the register data, was that EF and hence HFpEF/HFrEF status could not be accounted for. As there is no clear evidence for RASb treatment in HFpEF, guidelines do not recommend this treatment, and thus true eligibility for RASb for the patients in our cohort was not fully elucidated. This is a limitation for the interpretation of inequity in RASb access.

Disease severity was not measured, and may have confounded the association between employment and death, i.e., the non-employed patients could be non-employed for reasons related to a more severe form of HF. Sick leave/disability pension were not distinguishable from other types of non-employment in our data, and persons on sick leave due to severe HF would be classified as non-employed. Moreover, individuals might fail to obtain or retain a job due to poor health or disease severity-related factors. Thus, health selection was another potential methodological issue. However, the way patients were selected should mitigate the lack of disease severity data. Firstly, all patients were hospitalised for HF, indicating a similar disease severity, and they had a HF hospitalisation-free interval of at least one year before the index date. All included patients were also without RASb for 6 months prior to the index hospitalisation. Furthermore, those employed and those unemployed had similar hospitalisation-free time before index, indicating similar health care needs and comparable disease severity. We adjusted analyses for hospitalisation-free time. Therefore, although we lack data on HFpEF/HFrEF and disease severity, we believe that non-employed and employed patients should be fairly comparable in this regard, and confounding due to more severe HF consequently limited. Lastly, non-employment was registered in the year before the index date, thus preceding the index hospitalisation, which may mitigate the risk of health selection.

Comorbidity data was available only for the 1.5 years prior to the index date and based only on the ICD-10 codes of hospitalisations registered in the National Patient Register. Thus, comorbidity was likely underreported. We do not know if such underreporting was differential with respect to employment status or educational level. However, non-employed persons have been found to be more likely than the employed to abstain from seeking health care despite a need [13], which might increase the risk of underreporting.

Renal dysfunction is important as a possible confounder, as it is associated with higher mortality in HF [44] and may decrease the chance of receiving RASb. While renal dysfunction has been more prevalent in most other HF populations, our cohort was younger than most, and we excluded those with a recent RASb dispensation, which should lower the true proportion of renal dysfunction in the remaining cohort. Furthermore, our sensitivity analysis did not indicate any significant bias in our data due to underreporting of renal dysfunction.

### Clinical implications

Potential clinical implications of this study are that caregivers should consider socioeconomic disadvantage such as non-employment as a risk factor among HF patients, and adapt treatment accordingly. Closer follow-up may be appropriate. Health literacy could be an important factor, as well as psychiatric morbidity. Addressing health-related lifestyle risk factors might be especially important in this group. Health care has the potential to mitigate socioeconomic inequity in health. To inform such improvements in equity, research on the mechanisms behind these findings is needed. This would require data with more detailed clinical variables such as EF, and prescription data to assess drug adherence by socioeconomic factors.

## Conclusions

We have demonstrated that non-employment and low educational level were risk factors for elevated mortality among HF patients. Non-employment was associated with lower access to evidence-based treatment with RASb, which may constitute inequitable treatment; and access to RASb treatment was associated with a somewhat smaller risk reduction among the non-employed than among the employed. Non-employed HF patients thus appeared to have a higher death risk whether or not they received treatment.

## Supplementary Information

**Additional file 1 Table S1.** International Classification of Diseases (ICD-10) codes used to define comorbidity.

**Additional file 2 Table S2.** Patient characteristics among 30-day survivors by renin-angiotensin system blocker (RASb) dispensation within 30 days.

## Data Availability

The datasets generated and analysed during the current study are not publicly available due to the nature of the data, being sensitive personal information. The data, or parts thereof, may be available from the corresponding author on reasonable request from authorised persons. The registers from which the study data were obtained are not open to public access. The registers are held at The National Board of Health and Welfare (https://www.socialstyrelsen.se/en/statistics-and-data/registers/register-information/), and at Statistics Sweden (https://www.scb.se/en/services/ordering-data-and-statistics/guidance-for-researchers-and-universities/vilka-mikrodata-finns/longitudinella-register/longitudinal-integrated-database-for-health-insurance-and-labour-market-studies-lisa/)**.**

## Abbreviations

ACEI: Angiotensin-converting enzyme inhibitor
ARB: Angiotensin receptor blocker
HF: Heart failure
HFpEF: Heart failure with preserved ejection fraction
HFrEF: Heart failure with reduced ejection fraction
HR: Hazard ratio
ICD-10: International Classification of Diseases, tenth revision
LISA: Swedish acronym for the Longitudinal Integration Database for Health Insurance and Labour Market Studies
OR: Odds ratio
RAS: Renin angiotensin system
RASb: Renin angiotensin system blockers
RERI: Relative excess risk due to interaction

## Footnotes

1 International Classification of Diseases (ICD-10) codes: I11.0, I13.0, I13.2, I42.0, I42.3–I42.9, I50.0, I50.1, I50.9.

2 Anatomical Therapeutic Chemical class codes: C09A and C09B for ACEI; C09C and C09D for ARB.

3 Converted from Swedish krona; rate from the Swedish central bank (Riksbanken); https://www.riksbank.se/sv/statistik/ accessed 14 September 2020.
