# Supplementary material for: Non-employment and low educational level as risk factors for inequitable treatment and mortality in heart failure: a population-based cohort study of register data
Source: BMC Public Health. 2021 Jun 2;21:1040. doi: 10.1186/s12889-021-10919-1 (PMC8170987; doi:10.1186/s12889-021-10919-1)
Supplement: Supplementary file 1 — Additional file 1 Table S1. International Classification of Diseases (ICD-10) codes used to define comorbidity. [file 12889_2021_10919_MOESM1_ESM.docx]

| **S Table 1** International Classification of Diseases (ICD-10) codes used to define comorbidity. | |
| --- | --- |
| **Comorbidity diagnosis** | **ICD codes** |
| Hypertension | I10–I15 |
| Diabetes mellitus | E10–E14 |
| Angina pectoris | I20 |
| Myocardial infarction | I21–I22, I25.2 |
| Renal dysfunction | N17–N19, I13.1, I12.0, I13.2 |
| Atrial fibrillation/flutter | I48.0–I48.4, I48.9 |
| Dementia | F00–F03 |
| Pacemaker | Z95.0, Z45.0 |
| Stroke | I60–I61, I63–I64, I69.0–I69.4, G45 |
| Vascular disease | I70–I73 |
| Rheumatic disease | M05.0–M05.3, M05.8–M05.9, M06.0–M06.4, M06.8–M06.9, M31.5, M32.0–M32.1, M32.8–M32.9, M33.0, M33.1–M33.2, M33.9, M34.0–M34.2, M34.8–M34.9, M35.1, M35.3, M36.0 |
| Lung disease | J4–J9 |
| Liver disease | K70–K77 |
| Anaemia | D50–D64 |
| Cancer | C chapter |
| Coronary artery bypass grafting, CABG | Z95.1, Z95.5 |
