# Supplementary material for: Non-employment and low educational level as risk factors for inequitable treatment and mortality in heart failure: a population-based cohort study of register data
Source: BMC Public Health. 2021 Jun 2;21:1040. doi: 10.1186/s12889-021-10919-1 (PMC8170987; doi:10.1186/s12889-021-10919-1)
Supplement: Supplementary file 2 — Additional file 2 Table S2. Patient characteristics among 30-day survivors by renin-angiotensin system blocker (RASb) dispensation within 30 days. [file 12889_2021_10919_MOESM2_ESM.docx]

| **S Table 2** Patient characteristics among 30-day survivors by renin-angiotensin system blocker (RASb) dispensation within 30 days. | | | | |
| --- | --- | --- | --- | --- |
| Number (%) Distribution (%) | | | | |
|  | Total |  | RASb | No RASb |
|  |  |  |  |  |
|  | 3731 |  | 2802 (75.1) | 929 (24.9) |
|  |  |  |  |  |
| Age (years) |  |  |  |  |
| Median | 57 |  | 57 | 58 |
| 25th percentile | 50 |  | 50 | 49 |
| 75th percentile | 61 |  | 61 | 61 |
|  |  |  |  |  |
| Gender |  |  |  |  |
| Women | 1183 (31.7) |  | 780 (27.8) | 403 (43.4) |
| Men | 2548 (68.3) |  | 2022 (72.2) | 526 (56.6) |
|  |  |  |  |  |
| Education, N with data | 3628 |  | 2737 (75.4) | 891 (24.6) |
| Compulsory school | 1182 (32.6) |  | 886 (32.4) | 296 (33.2) |
| Upper secondary school | 1747 (48.2) |  | 1327 (48.5) | 420 (47.1) |
| Post-secondary school | 699 (19.3) |  | 524 (19.1) | 175 (19.6) |
|  |  |  |  |  |
| Employment status, N with data | 3694 |  | 2784 (75.4) | 910 (24.6) |
| Employed | 1980 (53.6) |  | 1627 (58.4) | 353 (38.8) |
| Non-employed | 1714 (46.4) |  | 1157 (41.6) | 557 (61.2) |
|  |  |  |  |  |
|  |  |  |  |  |
| Comorbidity |  |  |  |  |
| Hypertension | 1070 (28.7) |  | 839 (29.9) | 231 (24.9) |
| Diabetes mellitus | 562 (15.1) |  | 390 (13.9) | 172 (18.5) |
| Angina pectoris | 207 (5.5) |  | 129 (4.6) | 78 (8.4) |
| Myocardial infarction | 408 (10.9) |  | 269 (9.6) | 139 (15.0) |
| Atrial fibrillation/flutter | 930 (24.9) |  | 732 (26.1) | 198 (21.3) |
| Pacemaker | 112 (3.0) |  | 56 (2.0) | 56 (6.0) |
| Stroke | 111 (3.0) |  | 60 (2.1) | 51 (5.5) |
| Renal dysfunction | 201 (5.4) |  | 97 (3.5) | 104 (11.2) |
| Vascular disease | 62 (1.7) |  | 39 (1.4) | 23 (2.5) |
| Rheumatic disease | 64 (1.7) |  | 39 (1.4) | 25 (2.7) |
| Lung disease | 543 (14.6) |  | 348 (12.4) | 195 (21.0) |
| Liver disease | 86 (2.3) |  | 47 (1.7) | 39 (4.2) |
| CABG | 184 (4.9) |  | 120 (4.3) | 64 (6.9) |
| Anaemia | 169 (4.5) |  | 111 (4.0) | 58 (6.2) |
| Cancer | 102 (2.7) |  | 62 (2.2) | 40 (4.3) |
| Dementia | 8 (0.2) |  | 6 (0.2) | 2 (0.2) |
| Psychiatric disease | 337 (9.0) |  | 217 (7.7) | 120 (12.9) |
|  |  |  |  |  |
| CABG, coronary artery bypass grafting. | | | | |
